# The risk of myocardial infarction and ischemic stroke in pelvic cancer surgery

**DOI:** 10.1186/s40959-026-00545-4

**Published:** 2026-07-16

**Authors:** Nicola Giudici, Johan Björklund, Renata Zelic, Per Henrik Vincent, Simone Scuderi, Peter Wiklund, Markus Aly, Olof Akre, Juhana Rautiola

**Affiliations:** 1 Department of Molecular Medicine and Surgery, Department of Pelvic Cancer, Karolinska Institutet, Karolinska University Hospital, Stockholm, Sweden; 2 Department of Urology, University Hospital Bern, Bern, Switzerland; 3 Department of Urology, Icahn School of Medicine at Mount Sinai, New York, USA; 4 Unit of Urology, Division of Oncology, Gianfranco Soldera Prostate Cancer Lab, IRCCS San Raffaele Scientific Institute, Vita-Salute San Raffaele University, Milan, Italy

**Keywords:** Laparoscopic surgery, Open surgery, Myocardial infarction, Ischemic stroke, Pelvic cancer, Postoperative complications, Minimally invasive surgery

## Abstract

**Background:**

Myocardial infarction and ischemic stroke are common causes of morbidity after major cancer surgery. Understanding the risks associated with both open and laparoscopic surgery is crucial in reducing postoperative morbidity and mortality.

**Methods:**

This nationwide cohort study included patients who underwent major cancer surgery in the pelvis from 2000 to 2015. The primary outcome was the incidence of myocardial infarction and ischemic stroke within 90 days postoperatively. Adjusted odds ratios (OR) were estimated using logistic regression adjusted for propensity score and risks by regression standardization.

**Results:**

A total of 22,619 patients operated with laparoscopic and 70,896 patients operated with open approach were included in the analysis. The crude 90-day cumulative risks of myocardial infarction and ischemic stroke were 0.66% and 0.48%, respectively. Laparoscopic surgery was associated with lower odds of myocardial infarction within 30 days (OR 0.39, 95% CI 0.23–0.62) and 90 days (OR 0.57, 95% CI 0.41–0.81) compared to open surgery. Similarly, an association was observed for ischemic stroke (OR 0.67, 95% CI 0.38–1.05 at 30 days; OR 0.69, 95% CI 0.45–0.80 at 90 days).

**Conclusions:**

Laparoscopic surgery is associated with reduced postoperative risk of myocardial infarction and ischemic stroke compared to open surgery in the treatment of pelvic cancers. These findings favor the broader adoption of minimally invasive techniques in clinical practice.

**Supplementary Information:**

The online version contains supplementary material available at https://doi.org/10.1186/s40959-026-00545-4.

## Background

Myocardial infarction (MI) and ischemic stroke (IS) are known severe complications and major causes of mortality after surgery. The incidence of perioperative MI and IS is around 1–2% after non-cardiac surgeries, but the risk may increase substantially after major surgical procedures [1–4]. Few studies have compared different surgical approaches, i.e. open vs. laparoscopic surgery, with respect to risk of postoperative MI or IS.

In abdominal surgery, laparoscopic techniques have significantly minimized incisional tissue trauma while also offering shorter recovery times, reduced postoperative pain, a lower risk of infections, and smaller scars. As a result, many surgical procedures historically performed with open approach are now done laparoscopically [5, 6]. This is especially true in pelvic surgery, where due to the angle imposed by the pelvic bones and narrow anatomy of the pelvis, open surgery causes extensive skin incisions and considerable traction stress. Nonetheless, only a few studies have addressed the association between choice of surgical technique (open or laparoscopic) and cardiovascular and cerebrovascular ischemic events [7, 8]. 

In this study, we aimed to assess the risks of MI and IS during the first three months after major surgery for pelvic cancers comparing open and laparoscopic approach.

## Methods

This is a nationwide, population-based, observational cohort study of patients who underwent transabdominal laparoscopic or open surgery for bladder, prostate, cervical, endometrial or rectosigmoid cancer. Patients were followed for 90 days postoperatively for the occurrence of MI and IS.

### Study population

The study cohort includes all patients diagnosed with bladder, prostate, cervical, endometrial or rectosigmoid cancer for which they underwent a major transabdominal surgical procedure, as recorded in the Swedish National Patient Register from 1987 to 2015. For patients undergoing surgery for colorectal cancer, we decided to include only those procedures that involved at least a part of the pelvic area. Diagnoses were defined using the International Classification of Diseases, 9th and 10th Editions (ICD-9, ICD-10), and surgical procedures using a nationally adapted version of the NOMESCO Classification. Specific definitions of diagnoses and procedures are detailed in the Supplementary Appendix (eTable 1). The exposure in this study was surgical approach, where all surgical procedures were classified as laparoscopic or open based on the procedural codes (eTable 2, Supplementary Appendix). The analyses were performed as treated, meaning if open conversion happened, the procedure was counted as open surgery. As laparoscopic technique became commonplace around the year 2000, we excluded patients operated before the 1st of January 2000. A detailed list of exclusion criteria and the number of excluded subjects is presented in the Supplementary Appendix (eFigure1).

### Follow-up and assessment of outcome

Patients were followed up using the Cause-of-Death Register and the Swedish Patient Register for the first occurrence of MI or IS 30 and 90 days from the date of surgery. MI was identified by the following codes: ICD-9: 410, 411 A, 411B, 411X, 429 F, 429G, 429 W, 423 A, 423, 429; ICD-10: I21x-I23x. IS was identified by: ICD-9: 433, 434, 436, 437G and 362D; ICD-10: I63x, H341 and I649. In validation studies, the sensitivity of the Patient Register is for MI and IS 91.5% and 96%, respectively [9, 10]. 

### Statistical analyses

The main analysis was performed by combining all cancer types. We calculated crude cumulative risks and risk differences 30 and 90 days after the surgery.

The association between the surgical approach and outcome was evaluated using logistic regression to obtain adjusted odds ratios (ORs). Given the rarity of outcomes, we adjusted for confounding by adding the logit of the probability of having laparoscopic surgery, i.e., the propensity score, in the outcome model. Thus, propensity score was used for dimension reduction. The propensity score was initially modelled as a function of all prespecified confounders (age, sex, comorbidities, other major surgery, cancer type, hospital volume, year of surgery and healthcare region (eTable 3, Supplementary Appendix)) in a logistic regression model. However, year of surgery and hospital volume were strong predictors of exposure which led to a poor overlap of propensity score between subjects who had laparoscopic vs. open surgery (eFigure 2, Supplementary Appendix). Consequently, year of surgery and hospital volume were excluded from the propensity score model. While we believe that year of surgery and hospital volume are confounders, it is possible that they are strongly associated with the exposure but weakly with the outcome. Adjustment for such variables can introduce some bias in the exposure effect estimate, but this bias tends to be small and needs to be balanced with the need to control residual confounding [11]. Given that in our study analyses with and without adjustment for the year of surgery and hospital volume gave very similar results [12], the outcome model was finally adjusted for propensity score, the year of surgery and the hospital volume. Age at surgery in the propensity score model, and year of surgery, hospital volume and the logit of the propensity score in the outcome model were transformed using orthogonalized restricted cubic splines. The marginal risks of outcome for patients who had laparoscopic and open surgery were estimated by regression standardization after the outcome model [13]. 

Confidence intervals for the conditional odds ratios and regression standardized marginal risks, risk differences and risk ratios were estimated by bootstrapping (*n* = 1000). In every bootstrap we repeated all the model-fitting steps, including propensity score estimation. We present percentile confidence intervals.

All data management and statistical analyses were performed in Stata version 16.1 and 17.1 (StataCorp, College Station, TX, USA).

### Sensitivity analysis

To evaluate if main analysis results were affected by confounding by indication, we repeated the above specified analyses by also adjusting for preoperative tumour stage. Since preoperative tumour stage information was not available for gynaecological cancers in our data, this analysis was performed only among bladder, prostate and rectosigmoid cancer patients.

Preoperative tumour stage was missing for 25.1% of bladder, 29.7% of rectosigmoid and 10.9% of prostate cancer surgery patients. When the chance of being a complete case does not depend on the outcome, conditional on all the measured confounders, a complete-case analysis based on the logistic regression should give unbiased results [14]. The chance of being a complete case in our data was not associated with the observed values of outcome (Supplementary Appendix, eTable 4). Thus, instead of using multiple imputation to deal with missing data, we used a complete-case approach. We first replicated the main analysis in a population restricted to subjects with a non-missing preoperative tumour stage. Finally, we modelled the outcome as a function of the logit of propensity score, year of surgery, hospital volume and the preoperative tumour stage.

To evaluate how the association of the exposure with the outcomes is affected by the history of outcomes, we repeated the main analysis by excluding patients with history of MI – for the analysis of MI, patients with history of IS – for the analysis of IS, and patients with history of both MI or IS – for the analysis of MI and IS. Furthermore, we repeated the main analysis by reweighting the population using the inverse of the propensity score and then modelling the conditional expectation of outcomes as a function of exposure, spline transformed year of surgery and spline transformed hospital volume. Also, to evaluate if the adjustment for the hospital volume by adding it in the outcome model sufficiently accounts for the effect of the clustering by hospital, we repeated the main analysis by fitting a mixed effects model with a random intercept for surgery hospital, under the assumption of the unstructured covariance for the random effects. Finally, to evaluate if uncertainty in exposure assignment due to conversion to open surgery affects study results, we repeated the main analysis by excluding all subjects with procedural codes, which indicated conversion to open surgery. These codes are specified in the (eTable2, Supplementary Appendix).

### Exploratory analysis

To evaluate if the association between the exposure and the outcome was heterogeneous across different cancer surgery groups or by the year of surgery, we repeated the main analysis by adding an interaction term between the exposure and (i) the cancer surgery type and (ii) the categorized year of surgery (2000–2005, 2005–2010 and 2010–2015).

## Results

A total of 22,619 patients operated with laparoscopic, and 70,896 patients operated with open approach between 2000 and 2015 were included in the analyses (Supplementary Appendix, eFigure 1). During the study period, the overall proportion undergoing laparoscopic surgery increased from under 1% in 2000 to 59% in 2015, with, however, a lot of variation among the different surgical categories (eFigure 4). The median age at surgery was 66 years (IQR 60–72), and 37% of the patients were female. The baseline characteristics are presented in Table 1 and cancer-specific characteristics in the Supplementary Appendix, eTable 5. For the whole cohort, the crude 30-day and 90-day cumulative risks of MI were 0.48% and 0.66%, respectively. The corresponding risks for IS were 0.31% and 0.48%, respectively.

**Table 1:** The characteristics of patients included in the study, by surgical approach

	Laparoscopic (*N* = 22,619)		Open (*N* = 70,896)	
	*N*	%	*N*	%
Cancer surgery type				
Bladder cancer	467	2,1	5 137	7,3
Prostate cancer	16 459	72,3	19 409	27,4
Cervical cancer	690	3,1	2 541	3,6
Uterus cancer	2 341	10,4	14 997	21,2
Rectosigmoid cancer	2 662	11,8	28 785	40,6
Age (median, IQR)	65	59, 69	67	60, 74
Sex, female	4 367	19,3	30 649	43,3
Other major surgery	619	2,7	4 518	6,4
Comorbidities				
Ischemic heart disease	1 044	4,6	4 705	6,6
History of ischemic stroke	102	0,45	682	0,96
Congestive heart failure	223	0,99	1 939	2,7
Cardiac arrhythmia	1 273	5,6	4 766	6,7
Valvular disease	236	1,0	945	1,3
Peripheral vascular disease	132	0,6	889	1,3
Renal disease	129	0,6	635	0,9
Chronic pulmonary disease	777	3,4	3 055	4,3
Hypertension	5 869	26,0	16 323	23,0
Diabetes mellitus	1 483	6,6	6 108	8,6
Anemia	138	0,6	1 556	2,2
Preoperative T-stage*				
T1	9 452	48,3	12 826	24,1
T2	6 263	32,0	11 551	21,7
T3	2 423	12,4	12 136	22,8
T4	145	0,7	2 331	4,4
Missing	1 305	6,7	14 487	27,2
30-days outcomes				
Myocardial infarction	23	0,1	425	0,6
Ischemic stroke	27	0,1	263	0,4
All-cause mortality	25	0,1	630	0,9
90-days outcomes				
Myocardial infarction	51	0,2	567	0,8
Ischemic stroke	41	0,2	404	0,6
All-cause mortality	56	0,3	1 334	1,9

* For gynecological cancers the preoperative T-stage was not available. The distribution of the preoperative T-stage is thus presented only for patients with prostate cancer, bladder cancer or colorectal cancer

**Fig. 1:**
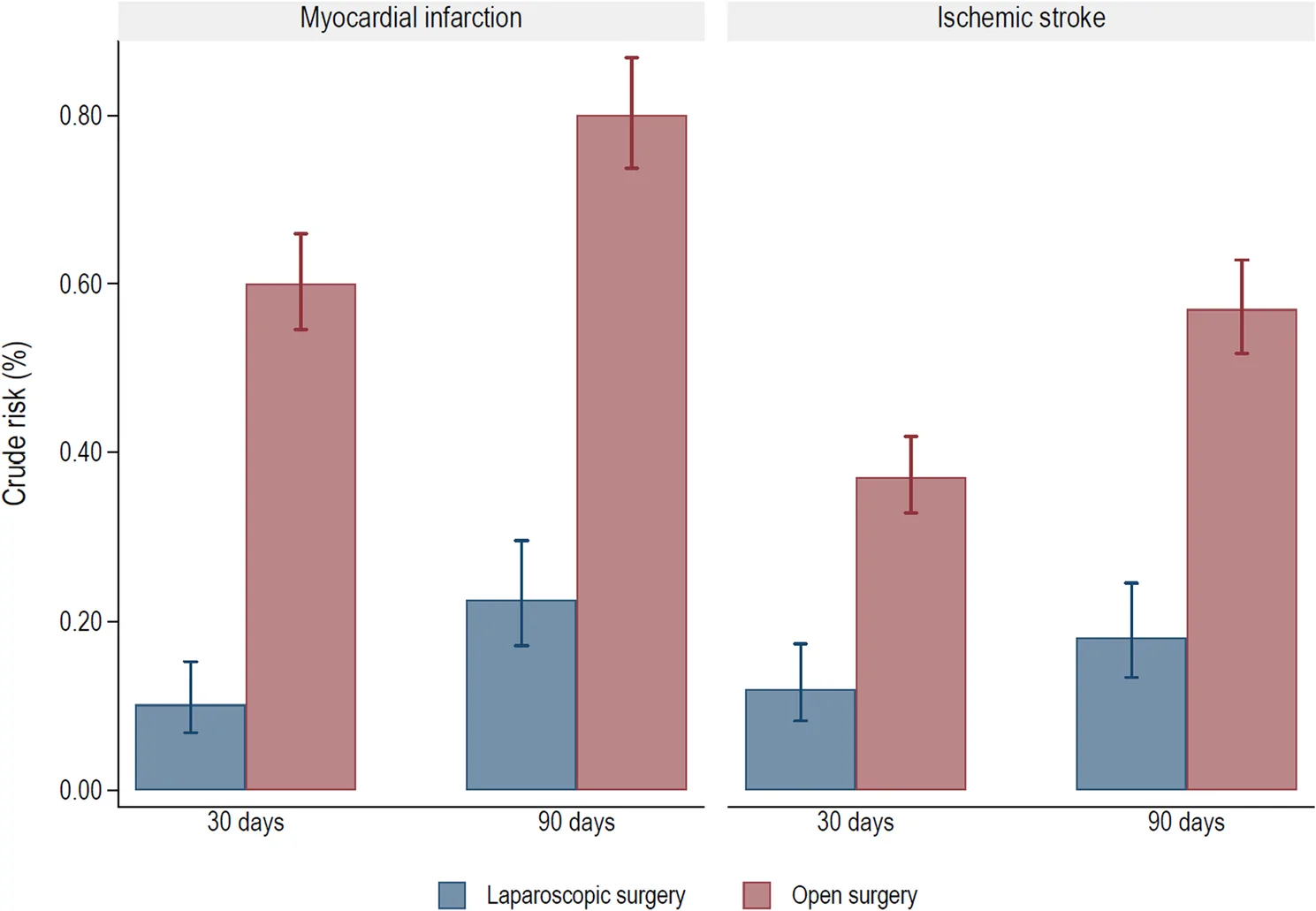
Crude cumulative risks of 30-day and 90-day myocardial infarction and ischemic stroke in patients who were operated for pelvic cancer using laparoscopic and open approach

The detailed cumulative risks by type of surgery are presented in Table 1; Fig. 1.

**Fig. 2:**
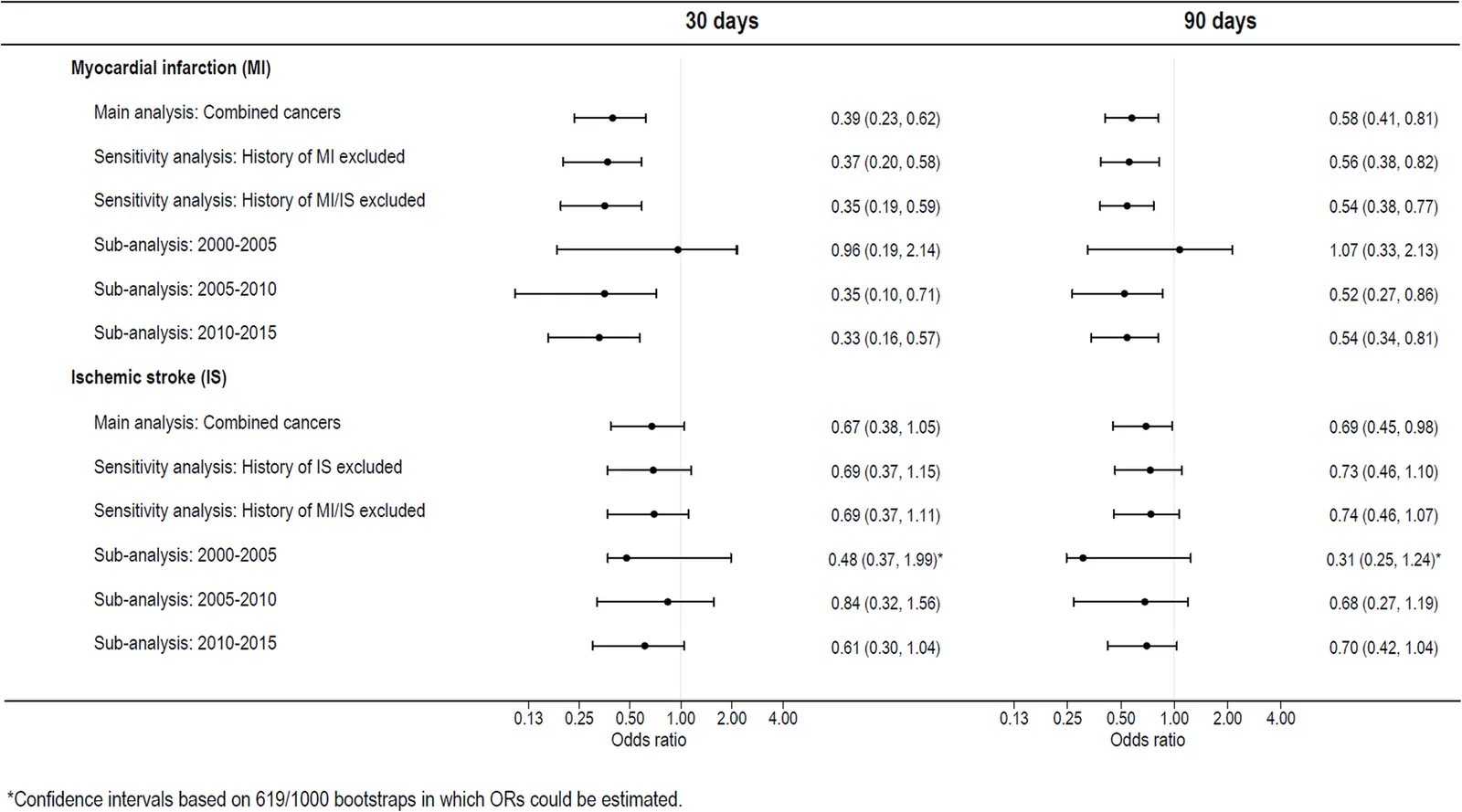
Adjusted odds ratios and 95% bootstrap percentile confidence intervals for the association of laparoscopic vs. open surgery and postoperative myocardial infarction and ischemic stroke

When adjusted for all the confounders, laparoscopic surgery was associated with lower odds of 30-day and 90-day postoperative MI compared to open surgery (OR 0.39; 95% CI 0.23–0.62 and 0.57; 95% CI 0.41–0.81, respectively). Similarly, laparoscopic surgery was associated with lower odds of IS at 30 days (OR 0.67; 95% CI 0.38–1.05) and 90 days (OR 0.69; 95% CI 0.45–0.98), respectively (Fig. 2).

### Sensitivity analysis

When the main analysis model was additionally adjusted for pre-surgery T-stage, we observed no changes in the estimates (Supplementary Appendix, eFigure 3, eTable 6). We further observed minimal changes in the estimates when we exclude subjects with the history of the outcome (Fig. 2), subjects who had conversion to open surgery code (Supplementary Appendix, eFigure 3), when confounding was adjusted using inverse probability of treatment weighting instead of adjustment for propensity score (Supplementary Appendix, eFigure 3, eTable 7) and when we added random intercept for surgery hospital in the mixed-effect model (eFigure 3).

### Sub-analysis

There was no indication of heterogeneity in the association between laparoscopic vs. open surgery with MI or IS across the different year of surgery categories or cancer surgery groups (Fig. 2, Supplementary Appendix, eFigure 3).

## Discussion

In this nationwide population-based study, we found that laparoscopic surgery was associated with a decreased risk of postoperative MI compared to open surgery in the first 30 and 90 days after surgery. Moreover, we found a similar, although not statistically significant reduced risk of ischemic stroke after laparoscopic surgery. Additionally, there was no heterogeneity of the association between surgery approach and MI or IS by the type of cancer surgery.

Major surgery is associated with severe and prolonged circulatory stress, which may trigger cardiovascular events. Furthermore, surgical trauma induces a systemic inflammatory response, leading to the release of inflammatory mediators, which can, in combination with surgery-related endothelial dysfunction and surgery-induced hypercoagulable state, promote plaque instability and arterial thrombosis. Moreover, perioperative physiological stress causes fluctuations in blood pressure and heart rate, which in turn can increase myocardial oxygen demand [15, 16]. As one of the main hypotheses for postoperative arterial ischemic events is the surgical trauma; in our study, all the analyses were performed as-treated. The pelvic organs are located deep within the pelvis, surrounded by bony structures. This tight space makes open surgery particularly invasive, requiring large incisions, extensive tissue manipulation and aggressive abdominal wall traction to access the operating field. In contrast, laparoscopic instruments can navigate this confined space with greater precision, reducing tissue damage and disruption. For these reasons, laparoscopy allows for a less invasive approach, leading to faster recovery, less physiological stress, decreased likelihood of significant blood loss and transfusions and reduced risk of wound infection, which is particularly advantageous in this scenario [17, 18]. In our study, there was no indication of heterogeneity in the association of surgical approaches with the outcomes in different cancer surgery groups. Thus, despite heterogeneous cancer groups, the same anatomical region with same fundamentals in surgical approach led to significant similarities in terms of postoperative arterial ischemic events.

To our knowledge, no study assessing the risk of arterial ischemic events following cancer surgery in the pelvis has been conducted. However, in non-cardiac surgery, a similar, or slightly higher, incidence of MI and IS has been demonstrated. This higher incidence may be related to the wider definition of ischemic events (for example troponin elevation) [19]. Smilowitz et al. demonstrated in a large cohort study, an in-hospital incidence of 1% for MI and 0.7% for IS in 10,581,621 non-cardiac surgeries. Cardiovascular events were more frequent in vascular (7.7%), thoracic (6.5%), and transplant surgeries (6.3%), explaining the overall higher rates compared to our study [3]. The potential impact of surgical approach on the risk of ischemic events was explored only in a few studies. Open cholecystectomy, for example, has been independently associated with an increased risk of MI and all-cause inpatient mortality when compared to laparoscopic cholecystectomy [7]. 

Beyond its strengths, our study entails several limitations. First, different sources of confounding may affect results from observational studies. While we adjusted all analyses for the extensive list of confounders, our results could still be affected by the residual confounding as not all information, such as information on disease stage, neoadjuvant therapies, or surgeon is available in the used registries. Furthermore, the study period encompasses a substantial shift from open to laparoscopic surgery, coinciding with significant advances in cardiovascular diagnostics and interventional cardiology, which may have introduced era bias and confounded the observed between-cohort differences in event detection, intervention rates, and cardiovascular mortality. Where possible, we sought proxies for the unavailable confounders, e.g., we used hospital volume as a proxy for surgical volume and experience, or we performed sensitivity analysis to evaluate robustness of the results. When we performed analyses additionally adjusted for preoperative tumor stage, the results were in line with our main analysis. However, it is possible that the disease stage is not sufficiently captured by preoperative tumor stage alone and N- and M-stage, which were inconsistently captured in the Swedish Cancer Register, have not been included in the analyses. Second, the accuracy of our results depends on the quality of data in healthcare registries, which can entail some misclassification of diagnoses. However, the Swedish in- and outpatient registers have been shown to be highly sensitive and specific regarding arterial ischemic events [9, 10]. Furthermore, the ICD codes used for the outcome definition do not capture the severity of myocardial infarction or ischemic stroke. Thus, the possible morbidity resulting from these outcomes is unknown. Third, the diagnosis and treatment of arterial ischemic events have improved during the time frame of our study, along with the shift from open surgery to laparoscopic surgery in certain cancer types. This might lead to overreporting of events in the laparoscopic cohort, which implies that the actual relative and absolute differences in risks may be even greater than reported. To account for this potential confounder, all the analyses were adjusted by the year of surgery.

## Conclusion

To our knowledge, this is the first study to investigate the risk of arterial ischemic events in the treatment of pelvic cancers and comparing laparoscopic and open approach. We found that laparoscopic surgery was associated with a reduced risk of myocardial infarction and ischemic stroke within the first three months after surgery. Our findings indicate a reduced risk of arterial ischemic events following minimally invasive surgery compared to open surgery in the treatment of pelvic cancers and underline the need for further development and implementation of these techniques in clinical practice.

## Supplementary Information

Supplementary Material 1.

## Data Availability

The datasets generated and analysed during the current study are not publicly available due ethical/privacy reasons, but anonymized quotes are available from the corresponding author on reasonable request.

## Abbreviations

MI: Myocardial infarction
IS: Ischemic stroke
ICD: 9–International Classification of Diseases, 9th Edition
ICD: 10–International Classification of Diseases, 10th Edition
NOMESCO: Nordic Medico–Statistical Committee
OR: Odds ratio
CI: Confidence interval
IQR: Interquartile range
