# Supplementary material for: The risk of myocardial infarction and ischemic stroke in pelvic cancer surgery
Source: Cardiooncology. 2026 Jul 16;12:125. doi: 10.1186/s40959-026-00545-4 (PMC13656610; doi:10.1186/s40959-026-00545-4)
Supplement: Supplementary file 1 [file 40959_2026_545_MOESM1_ESM.docx]

**Supplementary Online Content**

**The risk of myocardial infarction and ischemic stroke in pelvic cancer surgery**

Nicola Giudici^1,2^ M.D., Johan Björklund^1^ M.D., Ph.D., Renata Zelic^1^ M.D., Ph.D., Per Henrik Vincent^1^ M.ScE, Ph.D. Simone Scuderi ^1,5^ M.D., Peter Wiklund^1,3^ M.D., Ph.D., Markus Aly^1^ M.D., Ph.D., Olof Akre^1^, M.D., Ph.D. and Juhana Rautiola^1^, M.D., Ph.D.

^1^ Department of Molecular Medicine and Surgery, Karolinska Institutet and Department of Pelvic Cancer, Karolinska University Hospital, Stockholm, Sweden

2 Department of Urology, University Hospital Bern, Bern, Switzerland

^3^ Icahn School of Medicine at Mount Sinai, Department of Urology, New York, USA

^4^ Department of Pelvic Cancer, Karolinska University Hospital and Department of Women’s and Children’s Health, Karolinska Institutet, Stockholm, Sweden

5 Unit of Urology/Division of Oncology, Gianfranco Soldera Prostate Cancer Lab, IRCCS San Raffaele Scientific Institute, Vita-Salute San Raffaele University, Milan, Italy

**Table of contents**

[**eFigure1: Flow-chart of the population selection**	1](#_Toc225409914)

[**eFigure2: The distribution of propensity score among patients who had laparoscopic and open surgery**	2](#_Toc225409915)

[**eFigure 3. Adjusted odds ratios and 95% percentile confidence intervals for the association of laparoscopic vs. open surgery and postoperative myocardial infarction and ischemic stroke**	3](#_Toc225409916)

[**eFigure 4. Temporal Trends in the Use of Laparoscopic Surgery Overall and by Surgical Category (2000–2015)**	4](#_Toc225409917)

[**eTable 1. Diagnostic and procedural codes from the Swedish National Patient Register which were used for population definition**	5](#_Toc225409918)

[**eTable 2. The procedural codes from the Swedish National Patient Register that were used for the exposure definition**	6](#_Toc225409919)

[**eTable 3. The sources and Definition of the Confounders**	7](#_Toc225409920)

[**eTable 4. The missingness mechanism model for the complete case approach applied to subjects with non-missing preoperative tumor stage (N=72 919)**	14](#_Toc225409921)

[**eTable 5. The characteristics of patients included in the study, by cancer surgery type and surgical approach**	16](#_Toc225409922)

[**eTable 6. The adjusted odds ratios and regression standardized marginal risks, risk differences and risk ratios with the bootstrap percentile confidence intervals for the sensitivity analysis, the residual confounding by indication**	18](#_Toc225409923)

[**eTable 7. Sensitivity analyses with Inverse Probability Weighting (IPW)**	19](#_Toc225409924)

# **eFigure1: Flow-chart of the population selection**

N = 149 367

N = 93 488

| Exclusion reason: | N |
| --- | --- |
| 1. Index date before 2000 | 47 428 |
| 2. More than one cancer surgery on the index date | 578 |
| 3. Incomplete date of death in the COD^1^ | 162 |
| 4. Discharge date < index date | 0 |
| 5. Index hospitalization >90 days | 103 |
| 6. Surgery during emergency hospitalization | 7 352 |
| 7. Younger than 18 years of age | 6 |
| 8. Unreliable surgery codes for exposure definition^2^ | 250 |

^1^ Incomplete date of death refers to date records having 00 as the day and/or 00 as the month of death. If a matched comparison subject had an incomplete date of death in the Cause of death register, only that subject was excluded. If a cancer surgery patient had an incomplete date of death in the Cause of death register, that subject and all matched comparison subjects were excluded. A subject who died on the day 00 had to survive at least 1 year + 1 month from the index date not to be excluded. A subject who died on the month 00 had to survive a minimum of 1 year from the end of the index year not to be excluded.

^2^ Patients were excluded if during the index hospitalization they had records of:

1. Both open and laparoscopic procedure code AND diagnostic laparoscopy code (JAH01, JAP01) AND none of the exception codes (robot assisted surgery code: ZXC96, laparoscopy code: 4042, 4043, 4044, 4045, 4046, 4048, ZXC90, ZXC85, ZXC91) or conversion code (ZXK00 ZXK99))
2. (Only open procedure code OR both open and laparoscopic procedure code) AND laparoscopic LND code (8879, PJD63, PJD64, PJD97) AND none of the exception codes
3. (Only laparoscopic procedure code OR both open and laparoscopic procedure code) AND open LND code (PJD43, PJD44, PJD45, PJD53, PJD54, PJD55, PJD98, PJD99) AND none of the exception codes
4. Both open and laparoscopic procedure code AND none of the exception codes

# **eFigure2: The distribution of propensity score among patients who had laparoscopic and open surgery**

# **eFigure 3. Adjusted odds ratios and 95% percentile confidence intervals for the association of laparoscopic vs. open surgery and postoperative myocardial infarction and ischemic stroke**

# **eFigure 4. Temporal Trends in the Use of Laparoscopic Surgery Overall and by Surgical Category (2000–2015)**

# **eTable 1. Diagnostic and procedural codes from the Swedish National Patient Register which were used for population definition**

| **Cancer surgery type** | **Diagnostic codes^a^** | | **Procedural codes** | |
| --- | --- | --- | --- | --- |
|  | **ICD-9** | **ICD-10** | **K06** | **KVÅ** |
| Bladder | 188x | C67x | 6318, 6320-28, 6331 | KCC00, KCC10, KCC20, KCC30, KCC96 |
| Cervical | 180x | C53x | 7215-17, 7220-23, 7250-52, 7259 | LCD00-01, LCD04, LCD30-31, LCD96-97, LCE00, LCE10, LCE20, LCE96 |
| Endometrial | 179x, 182x | C54x, C55x |  |  |
| Prostate | 185 | C61x | 6611, 6631, 6633, 6639 | KEC00-01, KEC10, KEC20 |
| Rectosigmoid | 153x, 154x | C18x, C19x, C20x | 4644, 4652-54, 4810, 4820-23, 4828-29 | JFB46-47, JFB53-54, JFB60-61, JGB00-01, JGB03-04, JGB10-11, JGB20, JGB30-31, JGB33-34, JGB36, JGB40, JGB60-61, JGB96-97, JFH20, JFH30-31, JFH33, JFH40 |
| Abbreviations: ICD, International Classification of Disease (Swedish version); K06, The national classification for operations, sixth edition (1988); KVÅ, Klassifikation av vårdåtgärder, an adapted version of NOMESCO classification of surgical procedures  ^a^ ICD-9 and ICD-10 codes recorded as a main, or any of the 30 contributory diagnoses | | | | |

# **eTable 2. The procedural codes from the Swedish National Patient Register that were used for the exposure definition**

| **Cancer type** | **Procedural codes**^1^ | |
| --- | --- | --- |
|  | **K06** | **KVÅ** |
| Bladder | Open: 6318, 6320-28, 6331 | Open: KCC00, KCC10, KCC20, KCC30, KCC96 |
| Cervical | Open: 7220-23, 7250-52, 7259  Laparoscopic: 7215-17 | Open: LCD00, LCD30, LCD96, LCE00, LCE10, LCE20, LCE96  Laparoscopic: LCD01, LCD04, LCD31, LCD97 |
| Endometrial |  |  |
| Prostate | Open: 6611, 6631, 6633, 6639 | Open: KEC00, KEC10, KEC20  Laparoscopic: KEC01 |
| Rectosigmoid | Open: 4644, 4652-54, 4810, 4820-23, 4828-29 | Open: JFB46, JFB53, JFB60, JGB00, JGB03, JGB10, JGB20, JGB30, JGB33, JGB36, JGB40, JGB60, JGB96, JFH20, JFH30, JFH33, JFH40  Laparoscopic: JFB47, JFB54, JFB61, JGB01, JGB04, JGB11, JGB31, JGB34, JGB61, JGB97, JFH31 |
| ^1^ If any of the following codes is recorded, open surgery is considered laparoscopic (Robot assisted surgery: ZXC96; Laparoscopy: 4042, 4043, 4044, 4045, 4046, 4048, ZXC90). If a conversion code (ZXK00, ZXK99) is recorded among procedural codes, laparoscopic surgery is considered open.  2 If only laparoscopic procedure code OR both open and laparoscopic procedure code AND a conversion code (ZXK00 ZXK99) are present, the surgery is considered open  3 If only open procedure code OR both open and laparoscopic procedure code AND any of the following codes is recorded (robot assisted surgery: ZXC96 or laparoscopy: 4042, 4043, 4044, 4045, 4046, 4048, ZXC90, ZXC85, ZXC91) and no conversion (ZXK00 ZXK99) code is present, the surgery is considered laparoscopic  4 If only open procedure code and diagnostic laparoscopy code is recorded (JAH01 and/or JAP01) and none of the above specified exception codes are present, the surgery is considered open | | |

# **eTable 3. The sources and Definition of the Confounders**

| **Confounders** | **Source and definition** | **Time before index date** |
| --- | --- | --- |
| Comorbidities |  |  |
| Ischemic heart disease | NPR; ICD-9^1^: 410-413, 414A, 414B, 414W, 414X, 416X, 423A, 429C, 429F, 429G, 429W; ICD-10^1^: I20-I25 | < 1 year^2^ |
| Peripheral Vascular Disease | NPR; ICD-9^1^: 440, 441, 443, V43E; ICD-10^1^: I70, I71, I73 | < 1 year^2^ |
| Cardiac Arrhythmia | NPR; ICD-9^1^: 426, 427; ICD-10^1^: I44, I45, I47-I49, R00 | < 1 year^2^ |
| Non-hemorrhagic stroke | NPR; ICD-9^1^: 433A, 433B, 433C, 433D, 433W, 433X, 434A, 434B, 434X, 436, 437G, 362D, 438; ICD-10^1^: I63, I64, H341, I693, I694 | < 1 year^2^ |
| Valvular disease | NPR; ICD-9^1:^ 394, 395, 396, 397, 424, 746, V42C, V43D; ICD-10^1^: I05-I08, I34-I39, Q23 | < 1 year^2^ |
| Congestive Heart Failure | NPR; ICD-9^1^: 398, 414W, 422, 425, 428, 429X; ICD-10^1^: I42, I43, I50 | < 1 year^2^ |
| Chronic Pulmonary Disease | NPR; ICD-9^1^: 490-496, 501-505; ICD-10^1^: J40-47, J60-67 | < 1 year^2^ |
| Renal Disease | NPR; ICD-9^1^: 582, 583, 585, 586, 588, V42A, V56; ICD-10^1^: N03, N05, N18-19, N25, Z49 | < 1 year^2^ |
| Other major surgeries | NPR; KVÅ: AAA00, AAB00, AAB10, AAB20, AAB30, AAB99, AAC00, AAC05, AAC10, AAC15, AAC20, AAC30, AAC40, AAC99, AAD00, AAD05, AAD10, AAD15, AAD30, AAD40, AAD99, AAE00, AAE10, AAE20, AAE25, AAE30, AAE40, AAE50, AAE99, AAH10, AAH20, AAH30, AAH50, AAH60, AAH70, AAH80, AAH99, AAJ00, AAJ10, AAJ15, AAJ20, AAJ25, AAJ30, AAJ35, AAJ99, AAK00, AAK10, AAK50, AAK60, AAK80, AAK85, AAK99, AAL00, AAL10, AAL15, AAL20, AAL30, AAL40, AAL50, AAL99, AAM10, AAM30, AAM99, ABA00, ABB00, ABB02, ABB04, ABB10, ABB30, ABB40, ABB99, ABC10, ABC13, ABC16, ABC20-21, ABC23, ABC26, ABC28, ABC30, ABC33, ABC36, ABC40, ABC50, ABC53, ABC56, ABC60, ABC63, ABC66, ABC99, ABD10, ADA10, ADA20-21, ADA30-31, AWC00, AWE00, AWW99, BCA15, BCA20, BCA30-31, BCA40-41, BCA99, BDA10, BWA00, BWC00, BWE00, BWW99, DQB40, DQB50, EDB20, EJB30, EKB00, ENB20, ENB30, FAA00, FAA10, FAA96, FAB00, FAB10, FAB20, FAB30, FAB96, FAC00, FAC10, FAC20, FAC96, FAD00, FAD10, FAD96, FAE00, FAE10, FAE20, FAE30, FAE40, FAE50, FAE96, FAF00, FAF10, FAF20, FAF96, FAW96, FBA00, FBA10, FBA96, FBB00, FBB10, FBB20, FBB50, FBB96, FBC00, FBC10, FBC96, FBD00, FBD10, FBD20, FBD96, FBE00, FBE10, FBE20, FBE32, FBE35, FBE42, FBE96, FBF00, FBF10, FBF20, FBF96, FBG00, FBG10, FBG96, FBH00, FBH10, FBH96, FBJ00, FBJ10, FBJ96, FBK03, FBK13, FBK96, FBL00, FBL10, FBL20, FBL30, FBL40, FBL50, FBL96, FBM00, FBM10, FBM20, FBM30, FBM96, FBN00, FBN10, FBN96, FBW96, FCA20, FCA60, FCA70, FCA80, FCE00, FCE10, FCE20, FCE30, FCE40, FCE96, FDA00, FDA10, FDA96, FDB03, FDB20, FDB96, FDC00, FDC10, FDC20, FDC96, FDD00, FDD10, FDD13, FDD20, FDD96, FDF00, FDG00, FDG10, FDG96, FDH00, FDH10, FDH30, FDH40, FDH96, FDJ00, FDJ10, FDJ20, FDJ30, FDJ42, FDJ96, FDM00, FDM10, FDM11, FDM20, FDM96, FDW96, FEB00, FEB10, FEB51, FEB96, FEC00-01, FEC10, FEC96, FED00, FED03, FED10, FED96, FEE10, FEF00, FEF10, FEF20, FEF31, FEF96, FEW96, FFA10, FFA20, FFA30, FFB00, FFB10, FFB96, FFC00, FFC10, FFC50, FFC60, FFC96, FFD00, FFD20, FFD96, FFE00, FFE02, FFE10, FFE96, FFF00, FFF10, FFF20, FFF96, FFG00, FFG10, FFG20, FFG30, FFG96, FFH00, FFH10, FFH96, FFJ00, FFJ10, FFJ96, FFK10, FFK20, FFK96, FFL00, FFL10, FFL96, FFW96, FGA00, FGA10, FGA96, FGB00, FGB10, FGB96, FGC00, FGC10, FGC96, FGD00, FGD03, FGD10, FGD30, FGD40, FGD96, FGE00, FGE10, FGE20, FGE96, FGW96, FHA00, FHA10, FHA20, FHA96, FHB00, FHB10, FHB20, FHB30, FHB40, FHB50, FHB60, FHB70, FHB80, FHB96, FHC00, FHC10, FHC20, FHC30, FHC96, FHD00, FHD03, FHD10, FHD30, FHD96, FHE00, FHE10, FHE20, FHE30, FHE40, FHE96, FHF00, FHF10, FHF20, FHF30, FHF96, FHG00, FHG10, FHG20, FHG96, FHH00, FHH10, FHH20, FHH96, FHJ00, FHJ10, FHJ96, FHW96, FJB00, FJB10, FJB96, FJC00, FJC10, FJD00, FJD10, FJD20, FJD96, FJE00, FJE10, FJE20, FJE30, FJE96, FJF00, FJF10, FJF20, FJF96, FJW96, FKA00, FKA10, FKA20, FKA96, FKB00, FKB10, FKB96, FKC00, FKC10, FKC20, FKC30, FKC40, FKC50, FKC60, FKC96, FKD00, FKD10, FKD20, FKD96, FKW96, FLB00, FLB10, FLC00, FLC10, FLC96, FLD00, FLD10, FLD20, FLD30, FLD50, FLD96, FLE00, FLE10, FLE20, FLE96, FLF00, FLW96, FMA00, FMA10, FMA20, FMA96, FMB00, FMB10, FMB20, FMB96, FMC00, FMC10, FMC20, FMC96, FMD00, FMD10, FMD20, FMD30, FMD33, FMD40, FMD96, FMW96, FNA00, FNA10, FNA20, FNA96, FNB00, FNB20, FNB96, FNC10, FNC20, FNC30, FNC40, FNC50, FNC60, FNC96, FND10, FND20, FND96, FNE00, FNE10, FNE20, FNE96, FNF00, FNF10, FNF20, FNF30, FNF96, FNG00, FNG10, FNG20, FNG30, FNG96, FNH00, FNH10, FNH20, FNH96, FNJ00, FNJ10, FNJ96, FNK00, FNK10, FNK20, FNK96, FNW96, FPA00, FPA10, FPA20, FPA30, FPA40, FPA96, FPB96, FPC00, FPC10, FPC96, FPD00, FPD96, FPG10, FPH10, FPH20, FPW96, FQA00, FQA10, FQA20, FQA30, FQA40, FQA96, FQB00, FQB10, FQB20, FQB30, FQB96, FQW96, FWA00, FWC00, FWE00, FWF00, FWG00, FWG02, FWW96, FWW98, FXA00, FXA10, FXA13, FXA20, FXA96, FXB00, FXC00, FXD00, FXE00, FXF00, FXG00, FXH00, FXJ00, FXK00, FXN00, GAA97, GAB00, GAB10, GAB13, GAB20, GAB30, GAB96, GAC10-11, GAC20-21, GAC33-34, GAC37, GAC40-41, GAC43-44, GAC46-47, GAC53-54, GAC56-57, GAC96-98, GAD00, GAD03, GAD10, GAD96, GAE16, GAE20, GAE23, GAE26, GAE30, GAE40, GAE50, GAF00, GAF03, GAF10, GAF96, GAG10-11, GAG20-21, GAG30-31, GAG33, GAG36, GAG40, GAG50, GAG96-97, GAW96-97, GBA46, GBA50, GBA53, GBA96, GBC00, GBC03, GBC06, GBC10, GBC13, GBC96, GCA20, GCA26, GCA30, GCA40, GCA43, GCA50, GCA60, GCA70, GCA96, GDA00, GDA20-21, GDA30-31, GDA40-41, GDA96-97, GDB00-01, GDB10-11, GDB20-21, GDB96-97, GDC00-01, GDC10-11, GDC13, GDC20, GDC23, GDC26, GDC96-97, GDD00-01, GDD10-11, GDD20, GDD23, GDD26, GDD96-97, GDG00, GDG03, GDG10, GDG13, GDG30, GDG96, GDW96-97, GEA00, GEA10, GEA20, GEA96, GEB10, GEB20, GEB30, GEB40, GEB96, GEC10, GEC13-14, GEC20, GEC23-24, GEC96-97, GEW96, GWA00, GWC00, GWE00, GWF00, GWW96, HAB00, HAB40, HAB99, HAC10, HAC15, HAC20, HAC22, HAC25, HAC99, HAE00, HAE05, HAE10, HAE20, HAE99, HAF00, HWC00, HWE00, HWF00, HWW99, JAA13, JAA20-21, JAA23-24, JAA96-97, JAB00-01, JAB04, JAB10-11, JAB14, JAB20, JAB30, JAB40-41, JAB44, JAB50-51, JAB60-61, JAB70-71, JAB80-81, JAB84, JAB96-97, JAC10-11, JAC14, JAC20, JAC30, JAC40-41, JAC44, JAC50-51, JAC60-61, JAC70-71, JAC80-81, JAC84, JAC96-97, JAD10-11, JAD13, JAD20, JAD23, JAD30, JAD33, JAD40-41, JAD43, JAD47, JAD50-51, JAD60-61, JAD63, JAD67, JAD70-71, JAD73, JAD80-81, JAD84, JAD87, JAD96-97, JAE10-11, JAE20, JAE30, JAE40-41, JAE50-51, JAE60-61, JAE70-71, JAE80-81, JAE84, JAF11, JAF20, JAF30, JAF40-41, JAF50-51, JAF60-61, JAF70-71, JAF80-81, JAF84, JAF96-97, JAG00-01, JAG10-11, JAG20, JAG30, JAG40-41, JAG50-51, JAG60-61, JAG70-71, JAG80-81, JAG84, JAH00-01, JAH20-21, JAH30, JAH33, JAH40, JAK00-01, JAK03-04, JAK10, JAL00-01, JAL10-11, JAL20-21, JAL23, JAL30-31, JAL50-51, JAL96-97, JAM00, JAM10, JAP00-01, JAQ00, JAQ10, JAR10-11, JAR20, JAR30, JAR40-41, JAR50-51, JAR60-61, JAR70-71, JAR80-81, JAS10-11, JAS80-81, JAW96-97, JBA00-01, JBA20-21, JBB00-01, JBB10-11, JBB96-97, JBC00-01, JBW96-97, JCA00-01, JCA20, JCA60, JCA96-97, JCB00-01, JCC00, JCC10-11, JCC20, JCC30, JCC96-97, JCD00, JCD03, JCD10-11, JCD13, JCD20, JCD96-97, JCE00-01, JCE10, JCE20-21, JCE30, JCE33, JCE40, JCE50, JCE96-97, JCW96-97, JDA60-61, JDA63, JDB00-01, JDC00, JDC10-11, JDC20, JDC30, JDC40, JDC96-97, JDD00-01, JDD96, JDE00-01, JDE10, JDE20, JDE30-31, JDE96, JDF00-01, JDF10-11, JDF20-21, JDF40-41, JDF50-51, JDF60-61, JDF96-98, JDG00-01, JDG10-11, JDG96-97, JDH00, JDH40-41, JDH50, JDH60-61, JDH63, JDH70-71, JDW10-11, JDW96-97, JEA00-01, JEA10, JEW96-97, JFA00, JFA10, JFA17, JFA60, JFA63, JFA70-71, JFA73-74, JFA76, JFA80-81, JFA83-84, JFA86, JFA96-97, JFB00-01, JFB10, JFB13, JFB20-21, JFB30-31, JFB33-34, JFB40-41, JFB43-44, JFB46-47, JFB50-51, JFB53-54, JFB60-61, JFB63-64, JFB96-97, JFC00-01, JFC10-11, JFC20-21, JFC30-31, JFC40-41, JFC50-51, JFC60-61, JFD00, JFD03-04, JFD10, JFD13, JFD20, JFD23, JFD96, JFE00, JFE96, JFF00-01, JFF10-11, JFF13, JFF16, JFF20-21, JFF23-24, JFF26-27, JFF30-31, JFF40-41, JFF50-51, JFF60, JFF96-97, JFG00, JFG10, JFG20, JFG23, JFG26, JFG29-30, JFG33, JFG36, JFG40, JFG50, JFG53, JFG56, JFG60, JFG70, JFG73, JFG76, JFG80, JFG83, JFG86, JFH00, JFH01, JFH10-11, JFH20, JFH30-31, JFH33, JFH40, JFH96, JFJ00-01, JFJ96-97, JFK00-01, JFK10, JFK20, JFK96-97, JFL00, JFL10-11, JFM00, JFW96-97, JGB00-01, JGB03-04, JGB10-11, JGB20, JGB30-31, JGB33-34, JGB36, JGB40, JGB50, JGB60-61, JGB96-97, JGC00-01, JGC40, JGC96-97, JGW96-97, JJA00, JJA10, JJA20-21, JJA23-24, JJA30-31, JJA40-41, JJA43-44, JJA50, JJA96-97, JJB00-01, JJB10-11, JJB20-21, JJB30-31, JJB40-41, JJB50-51, JJB53-54, JJB60-61, JJB71, JJB96-97, JJC00, JJC10, JJC20, JJC30, JJC40, JJC50, JJC60, JJC96, JJW96-97, JKA00, JKA10- 11, JKA20-21, JKA96-97, JKB00-01, JKB11, JKB40, JKB96-97, JKC00-01, JKC10, JKC20, JKC30, JKC40, JKC50, JKC96-97, JKD00-01, JKD10, JKD20, JKD30, JKD40, JKD50, JKD96, JKF00, JKF96-97, JKW96-97, JLA00, JLA10, JLA21, JLB00, JLB10, JLC00, JLC10-11, JLC20, JLC30, JLC40, JLC50, JLC96, JLD00, JLD10, JLD20, JLE00, JLE03, JLE10, JLE16, JLE20, JLE30, JLE40, JLE50, JLE56, JLE96, JLW96-97, JMA00, JMA10-11, JMA20, JMB00-01, JMB10, JMW96-97, JWA00, JWC00, JWE00, JWF00-01, JWW96-97, JXA00, JXA06, JXA10, JXA13, JXA16, KAA00, KAC00-01, KAC20-21, KAD00-01, KAD10, KAD40, KAD51, KAD96-97, KAE10, KAH00, KAH30, KAH40-41, KAH50, KAH70, KAH80-81, KAH96-97, KAJ10, KAJ96, KAS00, KAS10, KAS20, KAS40, KAS50, KAS60, KAS96, KAW96, KBA00-01, KBC00, KBD00, KBE96, KBF00-01, KBH00, KBH10, KBH20, KBH30, KBH40, KBH50, KBH96-97, KBJ00, KBJ10, KBJ20, KBJ40, KBJ60, KBJ70, KBJ96, KBV00, KBV10, KBW96-97, KCA00, KCC00, KCC10, KCC20, KCC30, KCC96, KCD10, KCD20, KCD40, KCF00, KCH00, KCH10, KCH20, KCH30, KCH33, KCH50, KCH60, KCH96, KCJ10, KCJ20, KCJ96, KDG10, KDG20, KDG50, KDH30, KDH50, KDH70, KDH96, KEA00, KEC00-01, KEC10, KEC20, KED00, KEV50, KEW96, KFC00, KFC10, KFH00, KGC00, KGC10, KKA00-01, KKA31, KKB10, KKF00, KKW96, KWA00, KWC00-01, KWE00-02, KWF00-01, KWW96, KXK00, KXK03, KXK06, LAA01, LAA31, LAA97, LAB00-01, LAC00-01, LAC10-11, LAC20-21, LAC30-31, LAC96-97, LAD00-01, LAE10-11, LAE20-21, LAF00-01, LAF10-11, LAF20, LAF30, LAG00-01, LAG10-11, LAG20-21, LAG96-97, LAW96-97, LBC10-11, LBC20-21, LBC96-97, LBD00-01, LBE00-01, LBF30-31, LBF40-41, LBF50-51, LBF60-61, LBF70, LBF96-97, LBW96-97, LCB00-01, LCB10-11, LCB14, LCB96-97, LCC00-01, LCC10-11, LCC96-97, LCD00-01, LCD04, LCD10-11, LCD30-31, LCD40, LCD96-97, LCE00, LCE10, LCE20, LCE96, LCF00-01, LCG10-11, LCG20-21, LCG30-31, LCG40-41, LCG96-97, LEE10, LEE20, LEE30, LEE40, LEE96, LEF13, LEF20, LEF23, LEF41, LEF50-51, LEF96-97, LEG20, LFC00, LFC96, LFD00, LFD10, LFE20, LFE96, LWA00, LWC00-01, LWE00-01, LWF00-01, MCA00, MCA10, MCA20, MCA30, MCA33, MCA96, MCB00, MCC00, MCW96, MWA00, MWC00, MWE00-01, MWF00-01, MWW96, NAB90-96, NAC90-96, NAG39, NAG49, NAG59, NAG69, NAG79, NAG89, NAG99, NAJ19, NAJ39, NAJ49, NAJ69, NAJ79, NAJ89, NAJ99, NAN09, NAN19, NAN29, NAN99, NAR09, NAR29, NAR39, NAR69, NAR79, NAT19, NAT29, NAW69, NAW89, NBB09, NBB19, NBB29, NBB39, NBB49, NBB59, NBB69, NBB99, NBC09, NBC19, NBC29, NBC39, NBC49, NBC59, NBC69, NBC99, NBG09, NBG19, NBG29, NBH22, NBH72, NBH92, NBJ19, NBJ39, NBJ49, NBJ59, NBJ69, NBJ79, NBJ89, NBJ99, NBN09, NBN19, NBN29, NBN39, NBN49, NBN99, NBP29, NBQ01-03, NBQ29, NBQ99, NBR09, NBR19, NBR29, NBR39, NBR49, NBR59, NBR69, NBR79, NBR89, NBR99, NBU09, NBU19, NBU29, NBW69, NBW89, NCB09, NCB19, NCB29, NCB39, NCB49, NCB59, NCB99, NCC09, NCC19, NCC29, NCC39, NCC49, NCC59, NCC99, NCJ39, NCJ49, NCJ59, NCJ69, NCJ79, NCJ89, NCJ99, NEH09, NEH99, NEJ19, NEJ39, NEJ49, NEJ69, NEJ79, NEJ89, NEJ99, NEN09, NEN19, NEN29, NEN99, NEP29, NEQ19, NEQ29, NEQ99, NER09, NER19, NER29, NER39, NER49, NER59, NER69, NER79, NER99, NEW69, NEW89, NFB09, NFB19, NFB29, NFB39, NFB49, NFB62, NFB99, NFC09, NFC19, NFC20-23, NFC29, NFC30-33, NFC39-43, NFC49, NFC59, NFC99, NFG39, NFG49, NFG59, NFG99, NFH22, NFH72, NFH92, NFJ19, NFJ39, NFJ49, NFJ59, NFJ69, NFJ79, NFJ89, NFJ99, NFN09, NFN19, NFN29, NFN39, NFN49, NFN99, NFP29, NFQ09, NFQ19, NFQ29, NFQ99, NFR09, NFR19, NFR29, NFR39, NFR49, NFR59, NFR69, NFR79, NFR89, NFR99, NFS99, NFU09, NFU19, NFU39, NFU49, NFU89, NFW69, NFW89, NGB09, NGB19, NGB29, NGB39, NGB49, NGB53, NGB59, NGB99, NGC09, NGC19, NGC29, NGC39, NGC49, NGC53, NGC59, NGC99, NGH02, NGH22, NGH72, NGH92, NGJ19, NGJ39, NGJ49, NGJ59, NGJ69, NGJ79, NGJ89, NGJ99, NGN09, NGN19, NGN29, NGN39, NGN49, NGN99, NGP29, NGQ09, NGQ19, NGQ29, NGQ99, NGR09, NGR19, NGR29, NGR39, NGR49, NGR59, NGR69, NGR79, NGR99, NGU03, NGU09, NGU19, NGU49, NGU69, NGU89, NGW69, NGW89, NXW69, NXW89, PBB99, PHC23, PHC99, PJA10, PJD41-45, PJD52-55, PJD63-64, VAA00, VAA11, VAA22, VAA33, VAB00, VAB11, VAB22, VAB33, VAC48, VAG00, VAG11, VAG22, VAG33, VAH26, VAH37, VAJ00, VAJ11-14, VAJ16, VAJ19, VAJ22-24, VAJ29, VAJ33-35, VAJ39, VAK10-11, VAK20, VAK22, VAK30, VAK33, VAL00, VAL11, VAL22, VAL33, VAN00, VAN33, VAP00, VAP11, VAP22, VAP33, VAP48, VAQ00, VAQ11-14, VAQ22-24, VAQ26, VAQ33-35, VAQ37, VAT33, VAT48, VAU11, VAU22, VAU33, VAW00, VAW11, VAW22, VAW33, VBA00, VBA11, VBA22, VBA33, VBA44, VBB00, VBB11, VBB22, VBB33, VBB44, VBC00, VBC09, VBC33, VBE00, VBE11, VBE22, VBE33, VBE44, VBF11, VBF22, VBF33, VBF44, VBG00, VBG33, VBG44, VBH16, VBH26, VBH37, VBH56, VBJ00, VBJ12-15, VBJ23-25, VBJ34-35, VBK10, VBK20, VBK30, VBK40, VBL00, VBL11, VBL22, VBL33, VBL44, VBN00, VBN33, VBP00, VBP11, VBP22, VBP33, VBP44, VBQ00, VBQ12-16, VBQ23-26, VBQ34-36, VBR00, VBR11, VBR22, VBR33, VBR44, VBT00, VBT38, VBU00, VBU11, VBU22, VBU33, VBU44, VBW00, VBW11, VBW22, VBW33, VBW44, VCA00, VCA10-11, VCA22, VCA33, VCA44, VCA99, VCB00, VCB10-11, VCB22, VCB33, VCB44, VCB99, VCC00, VCC08, VCC10-11, VCC22-33, VCC44, VCC55, VCC99, VCE00, VCE10-11, VCE22, VCE33, VCE44, VCE99, VCF11, VCF22, VCF33, VCF44, VCF99, VCG00, VCG10-11, VCG22-23, VCG33, VCG44, VCG99, VCH11, VCH22, VCH33, VCH34, VCH44, VCH92, VCH99, VCJ00, VCJ10-11, VCJ17, VCJ19, VCJ22-23, VCJ26-27, VCJ29, VCJ33, VCJ39, VCJ44, VCJ59, VCJ99, VCK10, VCK20, VCK30, VCK33, VCK90, VCK99, VCL00, VCL10-11, VCL22, VCL33, VCL44, VCL99, VCN00, VCN10-11, VCN22-23, VCN33, VCN44, VCN99, VCP00, VCP10-11, VCP22-23, VCP33, VCP44, VCP99, VCQ00, VCQ10-11, VCQ22-23, VCQ33, VCQ44, VCQ99, VCR00, VCR10-11, VCR22, VCR33, VCR44, VCR99, VCS10-11, VCS22, VCS33, VCS44, VCS99, VCT08-09, VCU00, VCU10-11, VCU22-23, VCU33, VCU44, VCU99, VCW00, VCW10-11, VCW22-23, VCW33, VCW44, VCW99, VEA00, VEA11, VEA18, VEA22, VEA33, VEA38, VEA99, VEB00, VEB11, VEB18, VEB22, VEB33, VEB38, VEB44, VEB98, VEB99, VEC00, VEC11, VEC18, VEC22, VEC33, VEC38, VEC98-99, VED18, VED38, VED98, VEE00, VEE11, VEE18, VEE22, VEE33, VEE38, VEE44, VEE98-99, VEF11, VEF22, VEF33, VEF99, VEG00, VEG11, VEG18, VEG22, VEG33, VEG38, VEG44, VEG98-99, VEH11, VEH16, VEH22, VEH98, VEJ00, VEJ11, VEJ13-19, VEJ33-39, VEJ98-99, VEK10, VEK18, VEK20, VEK30, VEK38, VEK90, VEK98, VEL00, VEL11, VEL18, VEL22, VEL33, VEL38, VEL44, VEL98-99, VEN00, VEN11, VEN18, VEN22, VEN33, VEN38, VEN98, VEN99, VEP00, VEP11, VEP18, VEP22, VEP33, VEP38, VEP98-99, VEQ00, VEQ11, VEQ22, VEQ33, VEQ99, VER00, VER11, VER18, VER22, VER33, VER38, VER44, VER48, VER55, VER58, VER65, VER68, VER98-99, VET00, VET11, VET18, VET22, VET33, VET38, VET98, VET99, VEU00, VEU11, VEU18, VEU22, VEU33, VEU38, VEU44, VEU98, VEU99, VEW00, VEW11, VEW18, VEW22, VEW33, VEW38, VEW98-99, VGA00, VGA11, VGA23, VGA34, VGA45, VGA99, VGB00, VGB11, VGB23, VGB34, VGB45, VGB99, VGC00, VGC11, VGC23, VGC34, VGC45, VGC67, VGC99, VGE00, VGE11, VGE23, VGE34, VGE45, VGE99, VGF11, VGF23, VGF34, VGF45, VGF99, VGG00, VGG11, VGG23, VGG34, VGG45, VGG99, VGH39, VGH49, VGH99, VGJ00, VGJ11, VGJ19, VGJ23, VGJ29, VGJ34, VGJ39, VGJ45, VGJ49, VGJ99, VGK10, VGK20, VGK30, VGK40, VGK60, VGK90, VGL00, VGL11, VGL23, VGL34, VGL45, VGL99, VGN00, VGN11, VGN23, VGN34, VGN45, VGN67, VGN99, VGP00, VGP11, VGP23, VGP34, VGP45, VGP67, VGP99, VGQ00, VGQ11, VGQ23, VGQ34, VGQ45, VGQ67, VGQ99, VGR00, VGR11, VGR23, VGR34, VGR45, VGR67, VGR99, VGT00, VGT11, VGT23, VGT34, VGT45, VGT67, VGT99, VGU00, VGU11, VGU23, VGU34, VGU45, VGU67, VGU99, VGW00, VGW11, VGW23, VGW34, VGW45, VGW99, VHA00, VHA11, VHA21, VHA31, VHA99, VHB00, VHB11, VHB21, VHB31, VHB99, VHC00, VHC11, VHC21, VHC31, VHC99, VHE00, VHE11, VHE21, VHE31, VHE99, VHF11, VHF21, VHF31, VHF99, VHG00, VHG11, VHG21, VHG31, VHG99, VHJ00, VHJ11-16, VHJ21-24, VHJ31, VHJ99, VHK10, VHK20, VHK30, VHK90, VHL00, VHL11, VHL21, VHL31, VHL99, VHN00, VHN11, VHN21, VHN31, VHN99, VHP00, VHP11, VHP21, VHP31, VHP99, VHQ00, VHQ11, VHQ21, VHQ31, VHQ99, VHR00, VHR11, VHR21, VHR31, VHR99, VHS00, VHS11, VHS21, VHS31, VHS99, VHT00, VHT11, VHT21, VHT31, VHT99, VHU00, VHU11, VHU21, VHU31, VHU99, VHW00, VHW11, VHW21, VHW31, VHW99, VKA00, VKA11, VKA18, VKA22, VKA33, VKB00, VKB11, VKB18, VKB22, VKB33, VKC00, VKC11, VKC18, VKC22, VKC33, VKD18, VKE00, VKE11, VKE18, VKE22, VKE33, VKF11, VKF22, VKF33, VKG00, VKG11, VKG18, VKG22, VKG33, VKH18, VKH22-23, VKH33, VKJ00, VKJ11-13, VKJ15, VKJ18-19, VKJ22-24, VKJ29, VKJ33-34, VKJ39, VKJ44, VKK10, VKK18, VKK20, VKK22-23, VKK30, VKK33, VKK40, VKL00, VKL11, VKL18, VKL22, VKL33, VKN00, VKN11, VKN18, VKN22-23, VKN33, VKP00, VKP11, VKP18, VKP22-23, VKP33, VKQ00, VKQ11, VKQ18, VKQ22-23, VKQ33, VKR00, VKR11, VKR18, VKR22, VKR33, VKR44, VKS00, VKS11, VKS18, VKS22, VKS33, VKT00, VKT11, VKT18, VKT22, VKT33, VKU00, VKU11, VKU18, VKU22, VKU33, VKW00, VKW11, VKW18, VKW22, VKW33, VME30, VME40, VMG31, VMG41, VMJ31, VMJ41, VML30, VML40, VMN30, VMN40, VMP31, VMP41, VMQ31, VMQ41, VMR30, VMR40, VMU30, VMU40, VMW30, VMW40, VPA00, VPA10, VPA20, VPA90, VPB00, VPB10, VPB20, VPB90, VPC01, VPC11, VPC21, VPC91, VPE00, VPE10, VPE20, VPE90, VPG01, VPG11, VPG21, VPG91, VPH13, VPJ01, VPJ11, VPJ21, VPJ91, VPK11-12, VPK21, VPK91-92, VPL00, VPL10, VPL20, VPL90, VPN00, VPN10, VPN20, VPN90, VPP01, VPP11, VPP21, VPP91, VPQ01, VPQ11, VPQ12, VPQ21, VPQ91, VPR00, VPR10, VPR20, VPR90, VPU00, VPU10, VPU20, VPU90, VPW00, VPW10, VPW20, VPW90, VQA00, VQA10, VQA20, VQA90, VQB00, VQB10, VQB20, VQB50, VQB90, VQC01, VQC11, VQC21, VQC51, VQC91, VQE00, VQE10, VQE20, VQE90, VQF10, VQF20, VQF90, VQG01, VQG11, VQG21, VQG91, VQJ11, VQJ21, VQJ91, VQK11-12, VQK21-22, VQK52, VQK91-92, VQL00, VQL10, VQL20, VQL90, VQN00, VQN10, VQN20, VQN90, VQP01, VQP11-12, VQP21, VQP91, VQQ01, VQQ11, VQQ21, VQQ91, VQR00, VQR10, VQR20, VQR90, VQS00, VQS10, VQS20, VQS90, VQT00, VQT20, VQT50, VQT90, VQU00, VQU10, VQU20, VQU90, VQW00, VQW10, VQW20, VQW90, VSA00, VSA10, VSA20, VSE00, VSE10, VSE20, VSE90, VSF10, VSF20, VSG01, VSG11-12, VSG21, VSJ11-12, VSJ21, VSK12, VSP01, VSP11, VSP13, VSP21, VSQ01, VSQ11, VSQ21, VSR00, VSR10, VSR20, VSR90, VSU00, VSU10, VSU20, VSW00, VSW10, VSW20, VSW90, VUC11, VUC21, VUC31, VUC41, VUC51, VUC91, VUH12, VUK14, VUK24, VUK34, VUL10, VUL20, VUL30, VUL40, VUL50, VUL90, VWA00, VWC00, VWE00, VWF00, VWG00, VWH00, VWW00, VXK11, VXK16, VXK21, VXK26, VXK31, VXK36-37, VXK41, VXK46-47, VXK51, VXK56, VXK61, VXK66-67, YGA10, YGA20, YJA00, YKA00-01, ZZN00, ZZN10, ZZN20, ZZP00, ZZP10, ZZP20, ZZR05, ZZR10, ZZR20, ZZR30, ZZR40, ZZR45, ZZR60, ZZS00, ZZS10, ZZS20; K06: 0051-53, 0100-01, 0109, 0111, 0130, 0140-42, 0190-93, 0199-202, 0209-10, 0219, 0234, 0244-46, 0248-301, 0309, 0311, 0320, 0343, 0350, 0360-61, 0800, 0830-31, 0900-02, 0910, 0919-22, 0939-43, 0950, 095-60, 0995-97, 2220-24, 2228-29, 2765-67, 2769, 2820-22, 2829-30, 2840-41, 2850-51, 2859, 2872, 3002-07, 3009-16, 3018, 3022-56, 3058, 3060-80, 3084-86, 3088, 3091-93, 3095, 3099-109, 3111-18, 3120-46, 3148-51, 3155, 3158-59, 3162-63, 3191, 3195-99, 3301, 3340-43, 3349, 3400-06, 3412-13, 3420, 3425-28, 3430-31, 3440-42, 3444, 3492, 3498-99, 3501-02, 3510-11, 3520-21, 3530-35, 3539-43, 3550-53, 3559, 3588, 3590-91, 3594-99, 3802, 3811-14, 3816-17, 3820, 3830-32, 3850, 3896, 4010-11, 4030, 4042, 4045-46, 4071, 4080-81, 4099, 4130, 4190, 4199-205, 4207, 4210-13, 4240-44, 4250-51, 4261-63, 4270-73, 4280-81, 4290, 4299, 4410-26, 4429-30, 4432, 4434-35, 4439, 4450-54, 4459-66, 4468-78, 4484-85, 4499, 4501, 4510-11, 4517, 4520, 4599-601, 4610-11, 4621, 4630-31, 4640-44, 4648-54, 4660-61, 4663-65, 4668-71, 4700, 4710-13, 4720-21, 4730-36, 4739-41, 4750-54, 4759-60, 4769, 4770-72, 4781-83, 4789-99, 4810, 4820-23, 4840, 4843, 4845, 4849, 5101, 5110-13, 5119-22, 5129, 5180-81, 5190, 5199-202, 5212, 5219, 5220-24, 5229, 5280, 5299, 5300, 5302, 5304, 5306, 5309-13, 5319, 5330-31, 5339-41, 5350-53, 5356-57, 5359-69, 5502, 5510-17, 5519-21, 5530-32, 5539, 5580, 5590-93, 5599, 5610-11, 5685-87, 5693-99, 6000, 6010-11, 6020, 6030, 6040-44, 6050-56, 6059-60, 6070-72, 6077, 6079, 6090-92, 6110, 6120, 6200-03, 6210-13, 6220-23, 6230, 6240-42, 6250-52, 6254, 6258-63, 6290, 6299, 6301-04, 6311-14, 6318-31, 6340, 6351-54, 6357, 6392, 6399, 6440-41, 6600, 6610-11, 6630-33, 6639, 6641, 6702, 6740-42, 6752, 6790, 6931-33, 6995-98, 7000, 7010-12, 7020-23, 7030-33, 7040-43, 7049, 7080, 7090-91, 7093-97, 7099, 7100-01, 7110-12, 7120-23, 7140-49, 7190, 7193-94, 7196, 7199-200, 7210-11, 7214-18, 7220-23, 7228-32, 7239-41, 7249-52, 7259, 7261-63, 7273-74, 7281, 7290-94, 7298-99, 7410, 7419, 7441, 7443-44, 7449, 7467, 7472, 7510, 7520-21, 7595-99, 7800, 7810-11, 7830, 7840, 7890, 7895-97, 8130-32, 8205-07, 8213-16, 8220-22, 8340-41, 8349, 8402, 8409-15, 8419, 8423-28, 8432-33, 8435, 8437-43, 8449, 8481, 8575, 8583, 8593, 8730-31, 8740-41, 8780-81, 8790-91, 8802-04, 8806-09, 8811-42, 8845-49, 8851-52, 8855-57, 8861-66, 8868, 8870-74, 8876, 8882-91, 8894-98, 8954, 8974-75 | < 1 year |
| Age at surgery | TPR; Categorized as ≤49, 50-59, 60-69, 70-79, ≥80 | At index date |
| Sex | TPR | At index date |
| Region of residence | TPR; Region of hospital in which index surgery was performed, where:  Region 1 = Blekinge län, Kristianstads län, Malmöhus län, Skåne län.  Region 2 = Hallands län, Göteborgs och Bohus län, Västra Götalands län, Älvsborgs län, Skaraborgs län, Värmlands län.  Region 3 = Östergötlands län, Jönköpings län, Kronobergs län, Kalmar län.  Region 4 = Stockholms län, Gotlands län.  Region 5 = Uppsala län, Södermanlands län, Örebro län, Västmanlands län, Dalarnas län/Kopparbergs län, Gävleborgs län.  Region 6 = Västernorrlands län, Jämtlands län, Västerbottens län, Norrbottens län. | At index date |
| Hospital Volume | NPR; The average number of procedure-specific surgeries (see eTable 1 for procedural codes) performed by each hospital in each calendar year, including the four years preceding the index year. The average was calculated based on the years in which the hospital had a record in the NPR. Bladder and prostate cancer surgeries were grouped into one procedure, all gynecological surgeries (including ovarian cancer: K06: 7010-12, 7020-23, 7030-33; KVÅ: LAD00-01, LAE10-11, LAE20-21, LAF00-01, LAF10-11, LAF20, LAF30,) were grouped into another procedure and colorectal cancer surgeries were grouped separately. | At index date |
| Calendar year of surgery | NPR | At index date |
| Pre-surgery tumor stage^3^ | NCR; Categorized as: T1 (T1, T1a, T1b, T1c, Tis, Ta) , T2 (T2, T2a, T2b, T2c), T3 (T3, T3a, T3b, T3c, T3d) and T4 (T4, T4a, T4b). | Before index date ^4^ |
| Abbreviations: NPR, The National Patient Register; ICD-10, International Classification of Disease (Swedish version); KVÅ, Klassifikation av vårdåtgärder, an adapted version of NOMESCO classification of surgical procedures; TPR, Total Population Register; NCR, National Cancer Register  ^1^ All the ICD codes in the National patient register were available as three-digit codes, except for the outcome related ICD codes which were available as four-digit codes  ^2^ Comorbidities were identified as the main or any contributory diagnoses from 1 year before the index date until, but not including, the index date. This definition likely led to underestimation of the incidence of chronic diseases commonly managed in primary care and do not require hospitalization or admission to outpatient care. It is, however, likely that such comorbidities could be recorded as one of the contributory diagnoses in NPR even when subjects were hospitalized for some unrelated reason. Since all cancer surgery patients were either hospitalized or admitted to the outpatient care on the index date, to allow for the non-differential misclassification of the confounders between subjects in the cancer surgery cohort and the comparison cohort, we did not include index date in the confounder definition.  ^3^ Tumor stage for gynecological cancers was not available in this data.  ^4^ The pre-surgery tumor stage was considered a record only if the diagnosis date in the NCR preceded the index date. To select the appropriate record when multiple entries existed for the same cancer, the tumor stage closest to the index date was prioritized. If multiple records shared the same diagnosis date, the entry with the highest tumor stage was selected, ensuring that benign records were excluded when non-benign options were available. | | |

# **eTable 4. The missingness mechanism model for the complete case approach applied to subjects with non-missing preoperative tumor stage (N=72 919)**

|  | **Myocardial infarction^1^** | **Ischemic stroke^1^** |
| --- | --- | --- |
|  | **OR (95% CIs)** | **OR (95% CIs)** |
| Outcome 90 days after the index date | 0.97 (0.74, 1.27) | 0.92 (0.66, 1.28) |
| Laparoscopic surgery | 1.11 (1.02, 1.22) | 1.11 (1.02, 1.22) |
| Age at the time of visit^2^ |  |  |
| orthogonalized basis 1 | 1.02 (0.99, 1.05) | 1.02 (0.99, 1.05) |
| orthogonalized basis 2 | 0.97 (0.95, 0.99) | 0.97 (0.95, 0.99) |
| orthogonalized basis 3 | 1.02 (0.99, 1.05) | 1.02 (0.99, 1.04) |
| orthogonalized basis 4 | 1.00 (0.98, 1.03) | 1.01 (0.98, 1.03) |
| standardized age | 1.05 (1.02, 1.07) | 1.05 (1.02, 1.07) |
| Year of surgery^2^ |  |  |
| orthogonalized basis 1 | 2.33 (2.25, 2.41) | 2.33 (2.25, 2.41) |
| orthogonalized basis 2 | 0.63 (0.61, 0.65) | 0.63 (0.61, 0.65) |
| orthogonalized basis 3 | 1.23 (1.20, 1.27) | 1.23 (1.20, 1.27) |
| orthogonalized basis 4 | 0.84 (0.82, 0.87) | 0.84 (0.82, 0.87) |
| standardized year | 0.18 (0.17, 0.19) | 0.18 (0.17, 0.19) |
| Hospital volume^2^ |  |  |
| orthogonalized basis 1 | 1.19 (1.15, 1.23) | 1.19 (1.15, 1.23) |
| orthogonalized basis 2 | 1.01 (0.99, 1.04) | 1.01 (0.99, 1.04) |
| orthogonalized basis 3 | 0.95 (0.93, 0.97) | 0.95 (0.93, 0.97) |
| orthogonalized basis 4 | 0.91 (0.89, 0.93) | 0.91 (0.89, 0.93) |
| standardized hospital volume | 1.36 (1.31, 1.40) | 1.36 (1.31, 1.40) |
| Health care region^3^ |  |  |
| Region 1 | 1.00 | 1.00 |
| Region 2 | 0.89 (0.82, 0.96) | 0.89 (0.82, 0.96) |
| Region 3 | 0.66 (0.60, 0.73) | 0.66 (0.60, 0.73) |
| Region 4 | 0.70 (0.65, 0.77) | 0.71 (0.65, 0.77) |
| Region 5 | 1.58 (1.46, 1.72) | 1.58 (1.46, 1.72) |
| Region 6 | 1.77 (1.60, 1.96) | 1.77 (1.60, 1.96) |
| Surgery for: |  |  |
| Bladder cancer | 1.00 | 1.00 |
| Rectosigmoid cancer | 1.22 (1.10, 1.36) | 1.22 (1.10, 1.36) |
| Prostate cancer | 0.37 (0.33, 0.40) | 0.37 (0.33, 0.40) |
| Sex | 1.09 (1.02, 1.16) | 1.09 (1.02, 1.16) |
| Lymph node dissection | 0.90 (0.82, 0.98) | 0.90 (0.82, 0.98) |
| Other major surgery | 1.12 (1.02, 1.24) | 1.12 (1.02, 1.24) |
| Comorbidities |  |  |
| Ischemic heart disease | 0.98 (0.88, 1.08) | 0.98 (0.88, 1.08) |
| History of non-hemorrhagic stroke | 1.11 (0.80, 1.52) | 1.10 (0.80, 1.51) |
| Congestive Heart Failure | 1.06 (0.91, 1.23) | 1.06 (0.91, 1.23) |
| Cardiac Arrhythmia | 1.07 (0.97, 1.18) | 1.07 (0.97, 1.18) |
| Valvular disease | 1.07 (0.87, 1.30) | 1.07 (0.87, 1.31) |
| Peripheral Vascular Disease | 0.92 (0.75, 1.15) | 0.92 (0.75, 1.15) |
| Renal Disease | 1.48 (1.17, 1.88) | 1.48 (1.17, 1.87) |
| Chronic Pulmonary Disease | 0.96 (0.85, 1.08) | 0.96 (0.85, 1.08) |
| Hypertension | 0.97 (0.91, 1.04) | 0.97 (0.91, 1.04) |
| Cerebrovascular Disease | 1.01 (0.82, 1.23) | 1.02 (0.82, 1.25) |
| Paralysis (Paraplegia and Hemiplegia) | 0.88 (0.57, 1.38) | 0.88 (0.57, 1.38) |
| Diabetes Mellitus | 0.97 (0.88, 1.06) | 0.97 (0.88, 1.06) |
| Anemia | 0.97 (0.82, 1.15) | 0.97 (0.82, 1.15) |
| ^1^ The missingness indicator was modelled in a logistic regression model conditional on the outcome, exposure and all the confounders  ^2^ Age at surgery, year of surgery and hospital volume were transformed using orthogonalized splines with 5 degrees of freedom.  ^3^ Region 1, Skåne (Malmöhus and Kristianstads), Blekinge; Region 2, Halland, Västra Götaland (Älvsborg and Skaraborg), Värmland; Region 3, Kronoberg, Kalmar, Jönköping, Östergötland; Region 4, Stockholm, Gotland; Region 5, Uppsala, Sörmland, Västmanland, Dalarna (Kopparberg), Gävleborg, Örebro: Region 6, Jämtland, Västernorrland, Norrbotten, Västerbotten | | |

# **eTable 5. The characteristics of patients included in the study, by cancer surgery type and surgical approach**

|  | **Bladder cancer** | | | | **Prostate cancer** | | | | **Cervical cancer** | | | | **Endometrial cancer** | | | | **Colorectal cancer** | | | |
| --- | --- | --- | --- | --- | --- | --- | --- | --- | --- | --- | --- | --- | --- | --- | --- | --- | --- | --- | --- | --- |
|  | **Lap** | | **Open** | | **Lap** | | **Open** | | **Lap** | | **Open** | | **Lap** | | **Open** | | **Lap** | | **Open** | |
|  | **N** | **%** | **N** | **%** | **N** | **%** | **N** | **%** | **N** | **%** | **N** | **%** | **N** | **%** | **N** | **%** | **N** | **%** | **N** | **%** |
| N | 467 | - | 5'137 | - | 16'459 | - | 19'409 | - | 690 | - | 2'541 | - | 2'341 | - | 14'997 | - | 2'662 | - | 28'785 | - |
| Age (median, IQR) | 69 | (63, 74) | 69 | (63, 75) | 64 | (59, 68) | 64 | (60, 68) | 42 | (37, 49) | 46 | (38, 58) | 68 | (61, 76) | 68 | (61, 76) | 70 | (62, 77) | 70 | (62, 78) |
| Sex, female | 117 | 25.1 | 1'227 | 23.9 | 0 | 0 | 0 | 0 | 690 | 100 | 2'541 | 100 | 2'341 | 100 | 14'997 | 100 | 1'219 | 45.8 | 11'884 | 41.3 |
| LND | 440 | 94.2 | 2'978 | 58 | 2'429 | 14.8 | 5'325 | 27.4 | 520 | 75.4 | 1'167 | 45.9 | 683 | 29.2 | 2'911 | 19.4 | 3 | 0.1 | 158 | 0.5 |
| Other major surgery | 21 | 4.5 | 296 | 5.8 | 361 | 2.2 | 537 | 2.8 | 28 | 4 | 91 | 3.6 | 79 | 3.4 | 517 | 3.4 | 130 | 4.9 | 3'077 | 10.7 |
| Comorbidities |  |  |  |  |  |  |  |  |  |  |  |  |  |  |  |  |  |  |  |  |
| Ischaemic heart disease (acute and chronic) | 41 | 8.78 | 536 | 10.43 | 663 | 4.03 | 736 | 3.79 | 10 | 1.45 | 36 | 1.42 | 118 | 5.04 | 709 | 4.73 | 212 | 7.96 | 2'688 | 9.34 |
| History of non-haemorrhagic stroke | 5 | 1.07 | 69 | 1.34 | 43 | 0.26 | 57 | 0.29 | 2 | 0.29 | 8 | 0.31 | 20 | 0.85 | 141 | 0.94 | 32 | 1.20 | 407 | 1.41 |
| Congestive Heart Failure | 13 | 2.78 | 152 | 2.96 | 88 | 0.53 | 103 | 0.53 | 1 | 0.14 | 20 | 0.79 | 41 | 1.75 | 348 | 2.32 | 80 | 3.01 | 1'316 | 4.57 |
| Cardiac Arrhythmia | 56 | 11.99 | 421 | 8.20 | 729 | 4.43 | 662 | 3.41 | 13 | 1.88 | 37 | 1.46 | 197 | 8.42 | 881 | 5.87 | 278 | 10.44 | 2'765 | 9.61 |
| Valvular disease | 6 | 1.28 | 101 | 1.97 | 152 | 0.92 | 137 | 0.71 | 1 | 0.14 | 7 | 0.28 | 26 | 1.11 | 161 | 1.07 | 51 | 1.92 | 539 | 1.87 |
| Peripheral Vascular Disease | 12 | 2.57 | 159 | 3.10 | 72 | 0.44 | 88 | 0.45 | 4 | 0.58 | 11 | 0.43 | 10 | 0.43 | 78 | 0.52 | 34 | 1.28 | 553 | 1.92 |
| Renal Disease | 11 | 2.36 | 129 | 2.51 | 77 | 0.47 | 69 | 0.36 | 0 | 0 | 9 | 0.35 | 14 | 0.60 | 77 | 0.51 | 27 | 1.01 | 351 | 1.22 |
| Chronic Pulmonary Disease | 54 | 11.56 | 418 | 8.14 | 468 | 2.84 | 519 | 2.67 | 13 | 1.88 | 76 | 2.99 | 117 | 5 | 474 | 3.16 | 125 | 4.70 | 1'568 | 5.45 |
| Hypertension | 188 | 40.26 | 1'651 | 32.14 | 4'086 | 24.83 | 3'530 | 18.19 | 51 | 7.39 | 186 | 7.32 | 768 | 32.81 | 3'750 | 25.01 | 776 | 29.15 | 7'206 | 25.03 |
| Cerebrovascular Disease | 13 | 2.78 | 143 | 2.78 | 148 | 0.90 | 199 | 1.03 | 3 | 0.43 | 15 | 0.59 | 50 | 2.14 | 305 | 2.03 | 86 | 3.23 | 971 | 3.37 |
| Paralysis (Paraplegia and Hemiplegia) | 3 | 0.64 | 26 | 0.51 | 13 | 0.08 | 23 | 0.12 | 1 | 0.14 | 4 | 0.16 | 8 | 0.34 | 39 | 0.26 | 12 | 0.45 | 154 | 0.54 |
| Diabetes Mellitus | 56 | 11.99 | 567 | 11.04 | 925 | 5.62 | 960 | 4.95 | 14 | 2.03 | 78 | 3.07 | 232 | 9.91 | 1'497 | 9.98 | 256 | 9.62 | 3'006 | 10.44 |
| Anemia | 4 | 0.86 | 54 | 1.05 | 22 | 0.13 | 43 | 0.22 | 9 | 1.30 | 53 | 2.09 | 23 | 0.98 | 322 | 2.15 | 80 | 3.01 | 1'084 | 3.77 |
| Pre-surgery T-stage |  |  |  |  |  |  |  |  |  |  |  |  |  |  |  |  |  |  |  |  |
| T1 | 146 | 31.26 | 1'346 | 26.20 | 8'987 | 54.60 | 9'805 | 50.52 | 0 | . | 0 | . | 0 | . | 0 | . | 319 | 11.98 | 1'675 | 5.82 |
| T2 | 172 | 36.83 | 1'874 | 36.48 | 5'447 | 33.09 | 5'349 | 27.56 | 0 | . | 0 | . | 0 | . | 0 | . | 644 | 24.19 | 4'328 | 15.04 |
| T3 | 17 | 3.64 | 380 | 7.40 | 1'090 | 6.62 | 777 | 4 | 0 | . | 0 | . | 0 | . | 0 | . | 1'316 | 49.44 | 10'979 | 38.14 |
| T4 | 9 | 1.93 | 192 | 3.74 | 9 | 0.05 | 20 | 0.10 | 0 | . | 0 | . | 0 | . | 0 | . | 127 | 4.77 | 2'119 | 7.36 |
| Missing | 123 | 26.34 | 1'345 | 26.18 | 926 | 5.63 | 3'458 | 17.82 | 0 | . | 0 | . | 0 | . | 0 | . | 256 | 9.62 | 9'684 | 33.64 |
| Outcome 30 days after the index date |  |  |  |  |  |  |  |  |  |  |  |  |  |  |  |  |  |  |  |  |
| MI | 1 | 0.21 | 52 | 1.01 | 11 | 0.07 | 34 | 0.18 | 0 | 0 | 2 | 0.08 | 1 | 0.04 | 44 | 0.29 | 10 | 0.38 | 293 | 1.02 |
| IS | 1 | 0.21 | 32 | 0.62 | 9 | 0.05 | 28 | 0.14 | 0 | 0 | 1 | 0.04 | 6 | 0.26 | 47 | 0.31 | 11 | 0.41 | 155 | 0.54 |
| All-cause mortality | 3 | 0.64 | 74 | 1.44 | 6 | 0.04 | 21 | 0.11 | 0 | 0 | 5 | 0.20 | 1 | 0.04 | 71 | 0.47 | 15 | 0.56 | 459 | 1.59 |
| Outcome 90 days after the index date |  |  |  |  |  |  |  |  |  |  |  |  |  |  |  |  |  |  |  |  |
| MI | 3 | 0.64 | 74 | 1.44 | 26 | 0.16 | 56 | 0.29 | 0 | 0 | 4 | 0.16 | 5 | 0.21 | 68 | 0.45 | 17 | 0.64 | 365 | 1.27 |
| IS | 1 | 0.21 | 51 | 0.99 | 16 | 0.10 | 39 | 0.20 | 0 | 0 | 4 | 0.16 | 9 | 0.38 | 82 | 0.55 | 15 | 0.56 | 228 | 0.79 |
| All-cause mortality | 9 | 1.93 | 237 | 4.61 | 13 | 0.08 | 35 | 0.18 | 1 | 0.14 | 10 | 0.39 | 7 | 0.30 | 174 | 1.16 | 26 | 0.98 | 878 | 3.05 |

# **eTable 6. The adjusted odds ratios and regression standardized marginal risks, risk differences and risk ratios with the bootstrap percentile confidence intervals for the sensitivity analysis, the residual confounding by indication**

| **Outcome** | **Time** | **Conditional odds ratios** | **Marginal risk (%)** | | **Marginal risk difference** | **Marginal risk ratio** |
| --- | --- | --- | --- | --- | --- | --- |
|  |  |  | **Open surgery** | **Laparoscopic surgery** |  |  |
|  |  | **OR (95% CIs)** | **Risk (95% CIs)** | **Risk (95% CIs)** | **RD (95% Cis)** | **RR (95% Cis)** |
| Analysis not adjusted for pre-surgery tumor stage^1,2^ | | | | | | |
| Myocardial infarction | 30 | 0.42 (0.21, 0.68) | 0.51 (0.45, 0.58) | 0.22 (0.12, 0.34) | -0.30 (-0.92, 0.01) | 0.42 (0.22, 0.68) |
|  | 90 | 0.59 (0.38, 0.87) | 0.71 (0.63, 0.79) | 0.42 (0.29, 0.58) | -0.29 (-0.91, 0.01) | 0.59 (0.38, 0.87) |
| Ischemic stroke | 30 | 0.78 (0.41, 1.28) | 0.31 (0.26, 0.37) | 0.24 (0.13, 0.38) | -0.07 (-0.35, 0.08) | 0.79 (0.41, 1.28) |
|  | 90 | 0.81 (0.48, 1.24) | 0.44 (0.38, 0.50) | 0.36 (0.22, 0.53) | -0.08 (-0.42, 0.08) | 0.81 (0.48, 1.28) |
| Analysis adjusted for pre-surgery tumor stage^1,2^ | | | | | | |
| Myocardial infarction | 30 | 0.42 (0.22, 0.68) | 0.51 (0.45, 0.58) | 0.22 (0.12, 0.34) | -0.30 (-0.42, -0.15) | 0.44 (0.22, 0.69) |
|  | 90 | 0.59 (0.38, 0.86) | 0.71 (0.63, 0.79) | 0.42 (0.29, 0.59) | -0.29 (-0.46, -0.09) | 0.59 (0.39, 0.87) |
| Ischemic stroke | 30 | 0.77 (0.41, 1.26) | 0.31 (0.26, 0.37) | 0.24 (0.13, 0.37) | -0.07 (-0.20, 0.08) | 0.77 (0.41, 1.26) |
|  | 90 | 0.81 (0.48, 1.23) | 0.44 (0.38, 0.51) | 0.36 (0.22, 0.52) | -0.08 (-0.24, 0.10) | 0.81 (0.48, 1.23) |
| Abbreviations: OR, Odds ratios; CIs, Confidence intervals; RD, Risk difference; RR, Risk ratio  ^1^ The analysis was restricted to subjects who had bladder, prostate and rectosigmoid cancer surgery and non-missing pre-surgery tumor stage (N=73 098)  ^2^ The models were adjusted for the logit of propensity score transformed using orthogonalized restricted cubic splines with 4 degrees of freedom and year of surgery and hospital volume both transformed using orthogonalized splines with 5 degrees of freedom. | | | | | | |

# **eTable 7. Sensitivity analyses with Inverse Probability Weighting (IPW)**

| t | Outcome | Adjustment | Conditional odds ratio |  |  | Marginal risk |  |  |  |  |  | Marginal risk difference |  |  | Marginal risk ratio |  |  |
| --- | --- | --- | --- | --- | --- | --- | --- | --- | --- | --- | --- | --- | --- | --- | --- | --- | --- |
|  |  |  |  |  |  | **Open surgery** |  |  | **Laparoscopic surgery** |  |  |  |  |  |  |  |  |
|  |  |  | **OR** | **95% CIs** |  | **Risk (%)** | **95% CIs** |  | **Risk (%)** | **95% CIs** |  | **RD** | **95% CIs** |  | **RR** | **95% CIs** |  |
| 30 | MI | spline logit of PS | 0.39 | 0.24 | 0.62 | 0.52 | 0.47 | 0.57 | 0.20 | 0.13 | 0.31 | -0.31 | -0.42 | -0.19 | 0.40 | 0.24 | 0.62 |
| 30 | MI | IPW | 0.44 | 0.22 | 0.77 | 0.50 | 0.45 | 0.56 | 0.22 | 0.12 | 0.38 | -0.28 | -0.42 | -0.11 | 0.45 | 0.22 | 0.77 |
| 30 | MI | SIPW | 0.39 | 0.19 | 0.67 | 0.52 | 0.46 | 0.58 | 0.20 | 0.11 | 0.34 | -0.31 | -0.45 | -0.16 | 0.39 | 0.19 | 0.67 |
| 30 | MI | OIPW | 0.39 | 0.22 | 0.66 | 0.35 | 0.29 | 0.40 | 0.13 | 0.08 | 0.21 | -0.21 | -0.30 | -0.11 | 0.39 | 0.22 | 0.66 |
| 30 | IS | spline logit of PS | 0.68 | 0.39 | 1.05 | 0.32 | 0.28 | 0.37 | 0.22 | 0.13 | 0.33 | -0.10 | -0.21 | 0.02 | 0.68 | 0.39 | 1.05 |
| 30 | IS | IPW | 0.81 | 0.35 | 1.61 | 0.31 | 0.27 | 0.37 | 0.25 | 0.12 | 0.48 | -0.06 | -0.23 | 0.18 | 0.81 | 0.35 | 1.61 |
| 30 | IS | SIPW | 0.72 | 0.34 | 1.32 | 0.33 | 0.28 | 0.38 | 0.23 | 0.12 | 0.40 | -0.09 | -0.23 | 0.09 | 0.72 | 0.34 | 1.32 |
| 30 | IS | OIPW | 0.65 | 0.35 | 1.07 | 0.23 | 0.19 | 0.28 | 0.15 | 0.09 | 0.23 | -0.08 | -0.17 | 0.02 | 0.65 | 0.35 | 1.07 |
| 90 | MI | spline logit of PS | 0.57 | 0.41 | 0.81 | 0.70 | 0.64 | 0.76 | 0.40 | 0.29 | 0.55 | -0.30 | -0.43 | -0.13 | 0.58 | 0.41 | 0.81 |
| 90 | MI | IPW | 0.53 | 0.34 | 0.78 | 0.70 | 0.64 | 0.77 | 0.38 | 0.25 | 0.55 | -0.33 | -0.49 | -0.15 | 0.54 | 0.35 | 0.79 |
| 90 | MI | SIPW | 0.51 | 0.32 | 0.74 | 0.71 | 0.65 | 0.78 | 0.36 | 0.24 | 0.52 | -0.35 | -0.50 | -0.18 | 0.51 | 0.33 | 0.74 |
| 90 | MI | OIPW | 0.54 | 0.37 | 0.79 | 0.50 | 0.44 | 0.57 | 0.27 | 0.20 | 0.38 | -0.23 | -0.34 | -0.10 | 0.54 | 0.37 | 0.79 |
| 90 | IS | spline logit of PS | 0.69 | 0.45 | 0.97 | 0.50 | 0.44 | 0.55 | 0.34 | 0.23 | 0.46 | -0.15 | -0.28 | -0.01 | 0.69 | 0.45 | 0.97 |
| 90 | IS | IPW | 0.68 | 0.38 | 1.14 | 0.49 | 0.43 | 0.55 | 0.33 | 0.20 | 0.52 | -0.16 | -0.33 | 0.06 | 0.68 | 0.38 | 1.14 |
| 90 | IS | SIPW | 0.65 | 0.38 | 1.00 | 0.50 | 0.44 | 0.56 | 0.32 | 0.20 | 0.48 | -0.18 | -0.33 | 0.00 | 0.65 | 0.38 | 1.00 |
| 90 | IS | OIPW | 0.62 | 0.39 | 0.91 | 0.36 | 0.31 | 0.41 | 0.22 | 0.15 | 0.31 | -0.14 | -0.24 | -0.03 | 0.62 | 0.39 | 0.91 |
